# Expression of AtMed15 of *Arabidopsis* in yeast causes flocculation and increases ethanol production in yeast culture

**DOI:** 10.1038/srep27967

**Published:** 2016-06-16

**Authors:** Pradeep Dahiya, Divya S. Bhat, Jitendra K. Thakur

**Affiliations:** 1 National Institute of Plant Genome Research, Aruna Asaf Ali Marg, New Delhi 110067, India

## Abstract

Mediator, a multiprotein complex involved in transcription of class II genes, was first discovered in yeast and then characterized in many metazoans revealing a striking structural conservation of the complex. However, sequences of Mediator subunits are not well conserved raising a question on the functional conservation of these individual subunits. In this study, expression of Med15 of *Arabidopsis* (AtMed15) in *gal11*∆ yeast could not complement the function of ScGal11 in galactose metabolism and resistance against cycloheximide. Surprisingly, AtMed15 changed the morphology of the yeast cells. The cells adhered strongly on the surface of the agar media, and showed robust flocculation in the liquid media without affecting the growth. The AtMed15-induced adhesion and flocculation were observed in different carbon sources. Calcium-assisted cell wall-bound mannan-binding proteins were found to be involved in this flocculation, which was unaffected by wide fluctuation of pH or temperatures revealing its constitutive robust nature. Expression of few flocculation related *Flo* genes was up-regulated in these cells. Interestingly, there was significant increase in ethanol production by the yeast expressing AtMed15. Robust and constitutive flocculation and increased ethanol production by yeast cells harbouring AtMed15 indicate an opportunity of its important usage in biotechnology industries.

In higher eukaryotes, transcriptional programme of a protein coding gene is dependent on the regulatory elements present within or outside the gene. These regulatory elements are recognized by specific transcription factors which have the ability to respond to specific developmental and environmental signals. The transcription factors perceive the signal and then communicate with the transcriptional machinery either directly or indirectly through the precisely orchestrated recruitment of cofactors^1^,^2^,^3^. The Mediator complex or Mediator is one such cofactor, and is required for basal and induced expression of almost all RNA polymerase II-dependent genes^4^,^5^,^6^,^7^. Mediator is a large complex made up of 25 or more subunits arranged in a way to provide modular interfaces for several intermolecular interactions^8^. Some of these subunits can interact with transcription factors whereas some other subunits can interact with components of RNA pol II transcriptional machinery, suggesting that Mediator functions as a communication bridge that can relay regulatory signals from transcription factors bound to the regulatory elements to the RNA pol II machinery bound to the promoter of the gene^4^,^6^,^9^,^10^. Combination of genetic, structural and biochemical studies of yeast and metazoan Mediator complexes suggest that the subunits of the complex form four distinct modules designated as head, middle, tail and kinase^1^,^4^,^8^,^11^,^12^. The head, middle and tail modules form the relatively stable core structure, whereas the kinase module associates reversibly with the core structure. It is suggested that the kinase module sterically blocks the RNA Pol II interactions with the core Mediator, and thus exert a repressive function^13^,^14^. The head and middle modules are involved in interactions with the core RNA Pol II machinery^15^,^16^, whereas subunits of the tail modules interact with DNA-bound transcription activators and repressors^17^,^18^,^19^,^20^,^21^.

The tail module includes the Med2, Med3, Med14, Med15 and Med16 subunits. The Med15 protein is an interesting example of a category of the Mediator subunits which have a large intrinsic disordered region^22^ and has evolved to process transcriptional signals for regulating distinct cellular processes. The *Med15* gene was initially identified in yeast as a positive regulator of the genes encoding proteins that are involved in galactose metabolism, and hence originally called *Gal11*^23^. The follow-up studies suggested that Gal11 is a general cofactor involved in transcriptional activation of many functionally unrelated genes^24^. We showed that the xenobiotic-activated Pdr1orthologs, and the fatty-acid-responsive Oaf1, interact with and require Gal11 as a critical coactivator to mediate ligand-dependent gene activation^19^,^20^. The activation domain of master regulator of galactose metabolism, Gal4 also target Gal11 for its function^25^,^26^. The mammalian Arc105 and worm Mdt-15, both Med15 orthologs, are targeted by sterol regulatory element-binding protein 1α (SREBP1α), which controls cholesterol and lipid homeostasis^21^. In *Caenorhabditis elegans*, the orphan nuclear hormone receptor 49 (NHR-49) interacts with Med15/Mdt-15 to regulate expression of genes involved in fatty acid and glucose homeostasis^27^. Moreover, Mdt-15 was also found to be involved in detoxification response pathway^27^. Together, these reports suggest that in eukaryotes ranging from simple unicellular yeasts to complex multicellular insects and mammals, Med15 is a node for diverse pathways relevant to processes like lipid metabolism, sugar metabolism and xenobiotic detoxification. In all these reports, Med15 has been shown to be targeted by the specific transcription factors to recruit transcriptional machinery at the cognate promoter to initiate the transcription process.

Bioinformatic analysis of all the Mediator subunits and biochemical purification of Mediator complex from *Arabidopsis* suggest that plant genomes also code for Med15^8^,^28^,^29^,^30^,^31^. In *Arabidopsis*, its role was discovered in salicylic acid-mediated defence response^32^. Recently in rice, based on the expression and SNP analyses, we suggested a role of Med15 in seed development^33^. We started this study with a curiosity to know if *Arabidopsis* Med15 functioned in same way as Gal11 in yeast. Surprisingly, expression of *Arabidopsis Med15* cDNA (*AtMed15*) in *Saccharomyces cerevisiae* could not complement the function of *Gal11* in yeast for drug resistance and galactose metabolism, but drastically changed the morphology of the cells. The yeast cells harbouring *AtMed15* showed robust flocculation in liquid medium and enhanced invasive growth and adhesiveness on solid medium. The flocculation could withstand wide fluctuation in pH and temperature. Moreover, there was significant increase in ethanol production by these yeast cells providing an important scope of possible biotechnological applications.

## Results

### AtMed15 cannot complement some functions of ScGal11 in yeast

**Figure 1:**
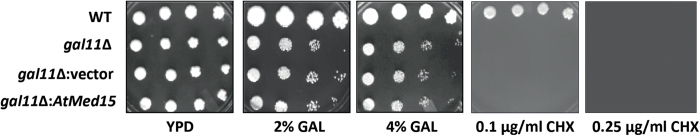
AtMed15 does not complement the function of Gal11 in yeast. Serially diluted equal amount of cells of WT, *gal11*∆, *gal11*∆ yeast carrying vector or vector harboring *AtMed15* cDNA were spotted on YP agar with galactose as carbon source or YPD agar with cycloheximide (CHX) as xenobiotic as mentioned under each panel. The plates were kept at 30 °C for 3–5 days.

Biochemical purification of Mediator complex from cultured cells, and bioinformatic analysis indicated presence of Med15 ortholog (At1G15780) in *Arabidopsis*^8^,^28^,^29^,^30^. Alignment of amino acid sequences of AtMed15 and Med15 from yeast and metazoans suggest that there is homology only in the amino terminal region which code for a KIX domain, whereas the other regions do not show noticeable similarity (Fig. S1). There are several other proteins which also contain KIX domain^31^,^33^,^34^. So, we designed functional complementation experiment to see if *AtMed15* was functionally similar to *ScGal11*. Yeast cells with mutated or without *Gal11* are not able to grow well in galactose medium^23^. The *AtMed15* cDNA was cloned in pGHM (pGHM was designed from pGADT7 by removing the Gal4 activation domain and HA tag from it) and introduced into *Scgal11*∆ strain, and then grown on media containing galactose as a carbon source. The transformed yeast cells carrying *AtMed15* cDNA behaved just like *Scgal11*∆ strain and *Scgal11*∆ harboring just the vector, and did not grow properly (Fig. 1). In yeast, master regulators of multidrug resistance, Pdr1 and Pdr3, require Gal11 for their transcriptional activity^20^. So, *Scgal11*∆ yeast cells are very sensitive to different drugs including cycloheximide (Fig. 1). As evident from the last two panels in the Fig. 1, the plant cDNA from *Arabidopsis* could not restore resistance to cycloheximide in the yeast. Both these results suggest that functionally AtMed15 is different from ScGal11, since former could not complement the function of later in yeast. Due to significant difference in the sequences (Fig. S1), AtMed15 might not be able to adopt the same configuration as ScGal11 in yeast.

### Expression of *AtMed15* induces flocculation and enhances adhesiveness of yeast

**Figure 2:**
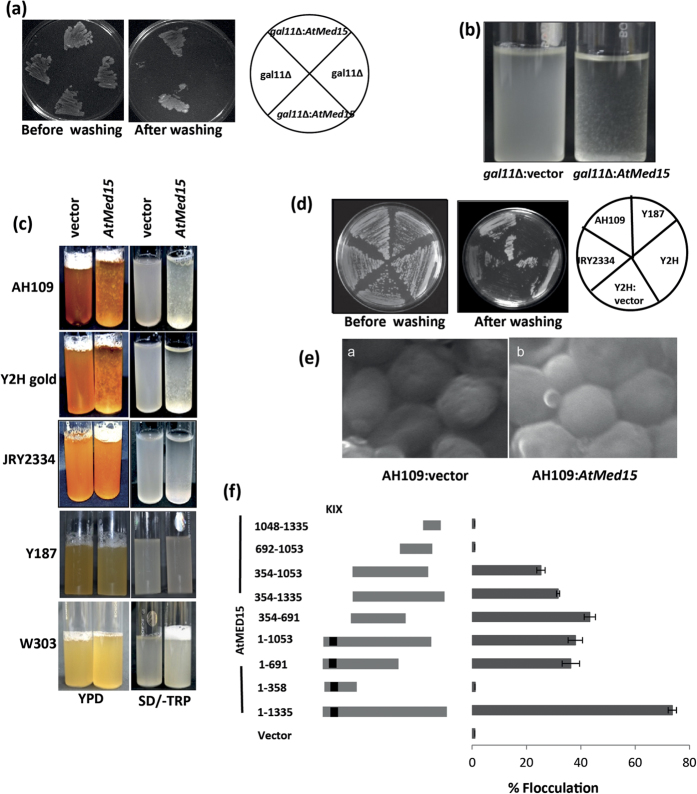
Expression of *AtMed15* in yeast increases adhesiveness of cells and causes flocculation. (**a**) Yeast cells as mentioned in the right panel, were streaked on YPD agar plate and grown for 3 days. The plate was photographed before (left) and after (middle) rinsing with water to wash off the loosely adhered cells. (**b)** Yeast cells as mentioned at the bottom, were grown in SD broth for 24 h to check flocculation. (**c)** Mat a (AH109, Y2H Gold and JRY2334) and Mat α (Y187 and W303) yeast cells transformed with vector or *AtMed15* were grown in liquid YPD or SD selection media for 24 h. (**d)** Different yeast strains (as shown in right panel) carrying *AtMed15* cDNA were streaked and grown on SD agar media. The plate was photographed before (left) and after (middle) washing. Y2H Gold strain with just the vector was used as a control. (**e)** Confocal microscopy of yeast with vector (left panel) and yeast carrying *AtMed15* (right panel). (**f)** Region of AtMed15 important for flocculation was characterized by analyzing the effect of deletion constructs on flocculation. AH109 yeast cells transformed with different fragments of *AtMed15* cDNA (left panel), were grown in YPD broth for flocculation analysis (right panel).

Expression of pGHM:*AtMed15* drastically changed the morphology of *Scgal11*∆ yeast cells. The cells became stickier and adhered to the surface of the media more strongly as assessed by the washing experiment, suggesting enhanced invasive growth in the agar media (Fig. 2a). Moreover, interestingly, these *Scgal11*∆ yeast cells with *AtMed15* cDNA displayed significant flocculation while growing in the liquid media (Fig. 2b). In order to know if the effect was because of *Gal11* deletion or vector specific, *AtMed15* was cloned in pGBKT7 and expressed in AH109 yeast strain. As AtMed15 was expressed with Gal4 DNA binding domain (DBD) present in pGBKT7, its activation property was checked for *His3* and *Ade2* which were under control of Gal4 promoter in AH109. AtMed15 did not induce expression of any of these reporter genes (Fig. S2a). Also, expression of Gal4 DBD-AtMed15 (fusion protein) did not affect the transcription of Gal4 target genes in yeast cells as assessed by the expression analysis of *Gal* genes (Fig. S2b). We observed similar changes in the phenotype of two more Mat a strains, like Y2H Gold and JRY2314, when transformed with *AtMed15*, suggesting the general ability of AtMed15 to affect the yeast cells irrespective of presence and absence of indigenous *Gal11* gene (Fig. 2c,d). On the other hand, expression of *AtMed15* in two Mat α type strains like Y187 and W303 did not show such robust changes (Fig. 2c,d). However, just on the basis of three Mat a and two Mat α strains used in this study, any conclusive relationship between the AtMed15-induced flocculation and mating type of yeast strains cannot be established. When yeast cultures were rotated for some time at slow speed, we observed that the Mat a yeast cells expressing *AtMed15* aggregated and formed clumps showing increased cell-cell adhesion (Fig. S3). This effect of AtMed15 in Mat α strain Y187 was very nominal (Fig. S3). There was no such aggregation and clumping in the vector control and untransformed cells (Fig. S3). Scanning electron microscopy of Mat a strains expressing AtMed15 showed dense packing of yeast cells within the flocs (Fig. 2e). Strong binding of neighbouring cells formed a very well defined tiling pattern of pressed cells with virtually no intracellular space (Fig. 2e). Thus, expression of *AtMed15* in yeast enhanced cell-cell adhesion and adherence on agar media, and induced flocculation and clumping in liquid media.

In order to find out which part of AtMed15 was responsible for flocculation in yeast culture, different fragments of this protein were expressed in the yeast cells. The carboxyl-end deletion of residues from 1054 to 1335 or 692 to 1335 reduced the flocculation to almost half but did not completely abolish it. Also, individual expression of these regions could not induce flocculation. The middle region 354–691 and large carboxyl side fragments 354–1053 and 354–1335 could induce similar level of flocculation (half of full-length AtMed15-induced flocculation), indicating the importance of 354–691 in flocculation. Thus, in AtMed15, the middle region of the protein spanning residues from 354 to 691 is required to induce flocculation in yeast cells (Fig. 2f). Surprisingly, none of the fragments could induce flocculation to the extent of full-length AtMed15. Region 1–358 harbours KIX domain which is known to be targeted by activation domain of several transcription factors^19^,^20^,^21^,^22^,^33^. Region 692–1053 carries four highly conserved signature sequence motifs (SSM 3 to SSM 7) of Med15 whereas region 1048–1335 harbours SSM 8^8^. These SSMs could be important for inter-subunit interactions and functional connections with PolII subunits or GTFs^8^. In this case, though the middle region of AtMed15 is required for flocculation, the other terminal regions (N-terminal KIX domain and C-terminal SSMs) are required by it to achieve its full potential. We think that transcriptional activators of flocculation might be targeting KIX domain of AtMed15 whereas SSMs of AtMed15 might be establishing contacts with other yeast Mediator subunits to help formation of functional PIC on the promoter of flocculin genes. As explained later, expression of AtMed15 in yeast indeed induce transcription of some flocculin genes.

### Factors affecting AtMed15-induced flocculation in yeast

**Figure 3:**
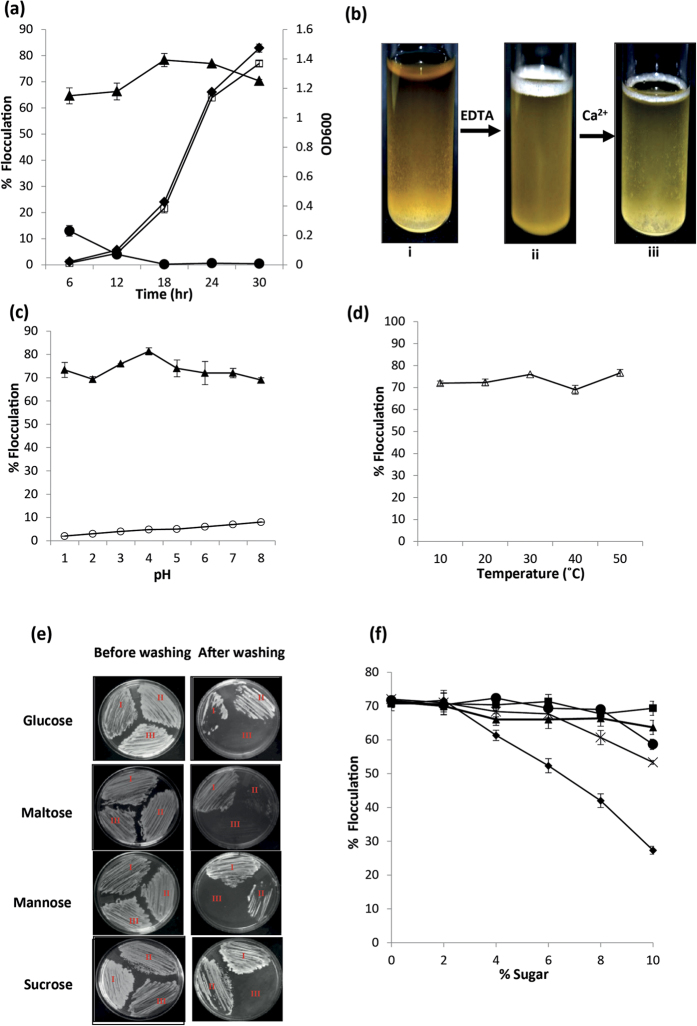
Effect of different factors on AtMed15-induced flocculation. (**a**) Cell growth and flocculation were evaluated in every 6 h at standard conditions, in SD^−^trp media, for AH109:pGBKT7 (yeast-vector) or AH109:pGBKT7-*AtMed15* (Yeast-*AtMed15*). (●) and (▲) indicate flocculation in vector and yeast-*AtMed15*, respectively, whereas (□) and (♦) represents growth of yeast-vector and yeast-*AtMed15*. (**b)** AH109:*AtMed15* yeast cells were grown in YPD broth (i), resuspended in buffer with EDTA (ii), and then resuspended in buffer with CaCl_2_ (iii). (**c)** Flocculation percentage was calculated for AH109 containing pGBKT7 vector (○) or pGBKT7:*AtMed15* (▲) grown over a broad range of pH (from 1 to 8). (**d)** Percentage of flocculation observed in AH109:*AtMed15* cells grown at 30 °C for 24 h and then transferred to different temperature ranging from 10 °C to 50 °C for 2 h. (**e)** Colonies of AH109:*AtMed15* (I), *gal11∆:AtMed15* (II) and Y187:*AtMed15* (III) were streaked and grown on solid media with different sugar as carbon source. The plates were photographed before (left panel) and after (right panel) rinsing with water to wash off the loosely adhered cells. (**f**) AH109:*AtMed15* yeast cells were first grown for 24 h in broth having glucose and then transferred to fresh media that had fructose (■), mannose (♦), maltose (×), sucrose (●) or glucose (▲) in it. Flocculation was measured in the fresh media after 8 h of growth at 30 °C.

Yeasts generally grow as dispersed cells when grown in liquid media. So, we analysed the growth curve of yeast cells to study the effect of AtMed15-induced flocculation on growth and found no effect on it (Fig. 3a). Cations play very important role in yeast flocculation; mostly they promote flocculation. The free and labile Ca^2+^ ions in the media enable cell wall bound lectins/mannoproteins adopt their active conformation, which in turn interacts with carbohydrate residues of neighbouring cells to cause flocculation^35^,^36^. In order to see effect of cations, EDTA was added in the culture media. Addition of EDTA caused dispersion of flocs in the flocculant yeast culture of AtMed15 expressing cells (Fig. 3b). The flocculation was reversible, as addition of excess CaCl_2_ in the medium could induce re-formation of flocs (Fig. 3b). Thus, AtMed15-induced flocculation in yeast was found to be dependent on Ca^2+^ ions. pH of the growth medium affects yeast flocculation. Some strains flocculate over a wide range of pH (2.5–9.0), whereas some brewing strains flocculate within a narrow pH range of 2.5–5.5^37^. The yeast cells with *AtMed15* cDNA were grown in YPD or SD media and then transferred to fresh media with the adjusted pH from 1.0 to 8.0. Flocculation was observed in all the tested pH (Fig. 3c). Temperature is considered to affect flocculation by breaking cell-cell interactions^38^. However, when we incubated *AtMed15* carrying yeast cells in liquid media at different temperatures for 8 hours, we did not see any noticeable difference in the extent of flocculation (Fig. 3d). A pH of 1.0 and temperature of 50 °C are lethal for yeast cells. In these conditions, less number of yeast cells was observed in the culture media, but whatever survived were able to form flocs (Fig. 3c,d). In all, there was no effect of pH and temperature on *AtMed15*-induced flocculation in yeast. Sugars have been known to affect flocculation by modulating cell-cell interactions^37^. We wanted to know if sugars as carbon source affected *AtMed15*-induced flocculation and adhesion. The yeast cells were grown in liquid and agar media with different carbon sources like glucose, maltose, sucrose, fructose or mannose, and flocculation in liquid broth and adhesiveness on solid agar surface were observed. In all these culture media, expression of AtMed15 could induce flocculation and enhance invasive growth in Mat a type yeast strains (Fig. 3e). Next, we transferred the yeast cells growing in glucose to fresh media containing different percentage of sugars in it. At higher percentage of mannose in the medium, flocs dispersed (Fig. 3f). This suggests that calcium-assisted mannan-binding proteins are involved in AtMed15-conferred flocculation in yeast.

### Yeast cell wall is not affected by AtMed15

**Figure 4:**
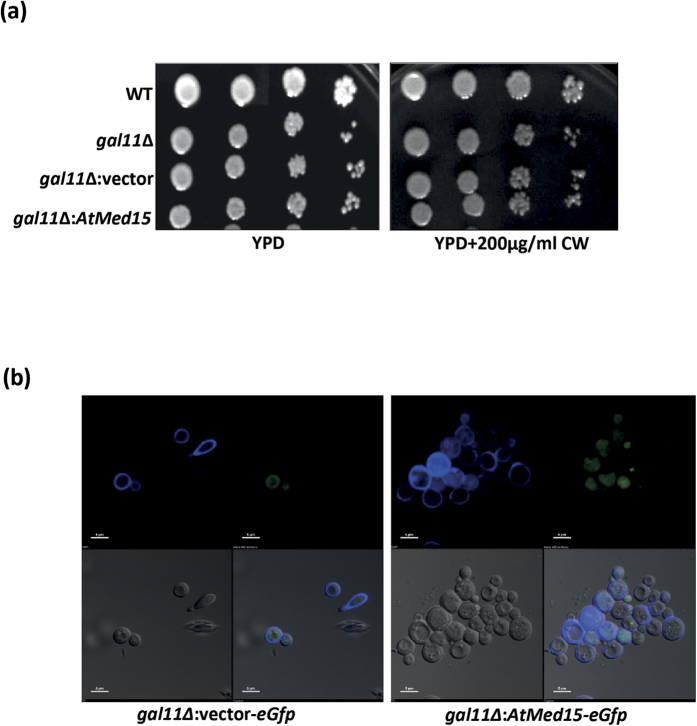
*AtMed15* is localized inside the nucleus and does not show any effect on the cell wall. (**a**) Serially diluted cells of WT, *gal11*∆, and *gal11*∆ transformed with vector or vector harboring *AtMed15* were spotted and grown on YPD or YPD with calcofluor white. (**b)** gal11∆ yeast cell having vector:*eGfp* or *AtMed15:eGfp* stained with calcoflour white were observed under confocal microscope to check the localization. Upper left panel represent imaging of calcofluor stained yeast cells under fluorescent light (excitation – 365 nm and emission – 420 nm). Upper right panel shows expression of eGfp under fluorescent light (excitation – 395 nm and emission – 509 nm). Lower left panel shows bright field imaging of yeast cells. The lower right panel shows superimposed image of all the three mentioned above.

Calcofluor White are used to stain the cell wall of yeast and other fungi^39^,^40^. Since cell-cell adhesion and flocculation are the properties which are directly related to the characteristics of cell wall, we wanted to see the effect of AtMed15 expression on yeast cell wall. First the yeast cells were tested for their sensitivity towards Calcofluor White. Expression of *AtMed15* in yeast cells did not affect the growth in the presence of Calcofluor White on the solid agar medium (Fig. 4a). Expression of AtMed15 can affect the cell wall related property by two ways; either it is expressed on the membrane to bring the change or it affects the expression level of genes that regulate the cell wall properties. In order to determine the localization of AtMed15 in yeast cells, *eGfp* was cloned in-frame downstream of *AtMed15* and cells were observed under confocal microscope. Expression of AtMed15 was found to be limited to nucleus only just like vector:*eGfp* control (Fig. 4b). This is not surprising as most of the transcriptional regulators are nuclear localized. So, next we proceeded to find the effect of AtMed15 on the expression of flocculation/adhesion related genes.

### Expression of AtMed15 in yeast affected expression of few *Flo* genes

**Figure 5:**
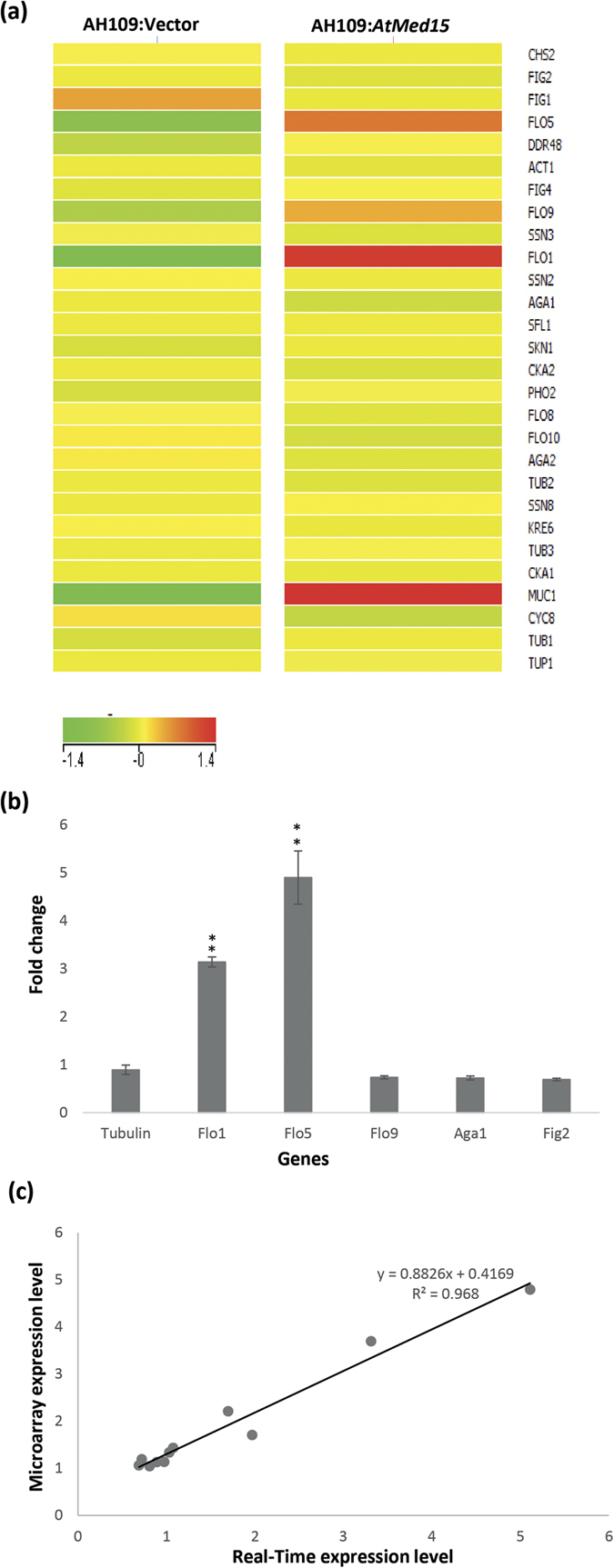
Expression of *AtMed15* increases transcript level of some flocculin genes. (**a**) Heat map of normalized and baseline transformed average log_2_ signal values of genes encoding for flocculins/adhesins in different yeast cells. The color legend is shown at the bottom and gene names are mentioned on the right of the heat map. Expression levels of *Act1* and *Tub1* were analyzed as controls. Yeast strains are mentioned on the top of the corresponding heat map. (**b**) Real-Time PCR analysis to determine the change in the transcript level of few selected flocculin/adhesin genes in AH109:*AtMed15* yeast cells as compared to AH109:Vector yeast cells. *Actin* was used as internal control. *Tubulin* was used as a comparison control for the expression analysis. Fold change represents expression level in AH109:*AtMed15* as compared to the control i.e. AH109:Vector. For, statistical analysis, student’s t-test was performed to determine the significance of difference. **indicate significance difference at p < 0.01. **(c)** Correlation of gene expression data obtained from Microarray analysis and Real-Time PCR analysis for 11 selected genes. The equation represents the correlation and R is correlation coefficient.

At least 33 genes are found to be associated with flocculation or cell aggregation processes in yeast^41^,^42^. The most important group of genes that are responsible for flocculation, code for flocculin or adhesion proteins *viz* Flo1, Flo5, Flo9 and Flo10^43^. Sequence wise, these proteins are very similar to each other. The second group of genes important for flocculation code for proteins like Flo11, Fig. 2 and Aga1^44^. These proteins are different from each other. The Flo8 protein is a transcription factor that regulates expression of many *Flo* genes^45^,^46^. In order to understand the mechanism of flocculation induced by AtMed15, we studied expression of flocculin and adhesion related genes in yeast cells harbouring *AtMed15* cDNA (pGBKT7-*AtMed15*) or just the vector (pGBKT7) as a control by microarray analysis and Real-Time PCR (Fig. 5a,b). The microarray data correlates very well with the quantitative Real-Time PCR analysis (Fig. 5c). Expression of *AtMed15* in AH109 yeast cells resulted in increased transcription of *Flo1, Flo5* and *Muc1* (Fig. 5a,b) while in *gal11*∆ yeast, it resulted in increased transcription of *Flo1*, *Flo11*/*Muc1* and *Aga1* genes in comparison to vector control and untransformed *gal11*∆ cells (Fig. S4a). Similar pattern of gene expression was observed in AH109 cells transformed with pGHM:*AtMed15* (Fig. S4b). For microarray analysis and corresponding Real-Time PCR analysis (for validation purpose) cells were harvested at 18 h (from the exponential phase) of the culture (Fig. 3a). In order to see the effect of AtMed15 on the expression of flocculin genes during the course of growth, we analysed the expression of few selected genes at 12 h and 24 h by Real-Time PCR (Fig. S5a,b). We also included *Gal* genes for comparison purpose. There was no difference in the expression profile of flocculin genes from 12 h to 24 h through 18 h spanning the exponential phase of the culture (Figs 5 and S5) As revealed by the gene expression analysis by microarray, deletion of *Gal11* caused reduction in the transcript level of some flocculin, adhesion and mannoprotein genes, suggesting that Gal11 is required for the expression of such genes and so might be an important factor to regulate flocculation and cell-cell adhesion in yeast (Fig. S4a). This is not surprising because Gal11 as a Mediator subunit functions as a co-regulator for many genes in yeast. We looked at all the genes of yeast being affected by expression of AtMed15 in the microarray analysis and tried to group them according to their functions. Microarray data was validated by quantitative Real-Time PCR analysis of a set of genes. There was a positive correlation between the two data with R^2^ value of 0.96 (Fig. 5c). GO analysis of the microarray data showed that the expression of AtMed15 in yeast cells mainly caused up-regulation of membrane related genes (Fig. S6). Thus, expression of *AtMed15* might be inducing flocculation in yeast culture partly by up-regulating the transcription of some selected flocculin/adhesin and mannoprotein coding genes.

### AtMed15 increased ethanol production in yeast culture

**Figure 6:**
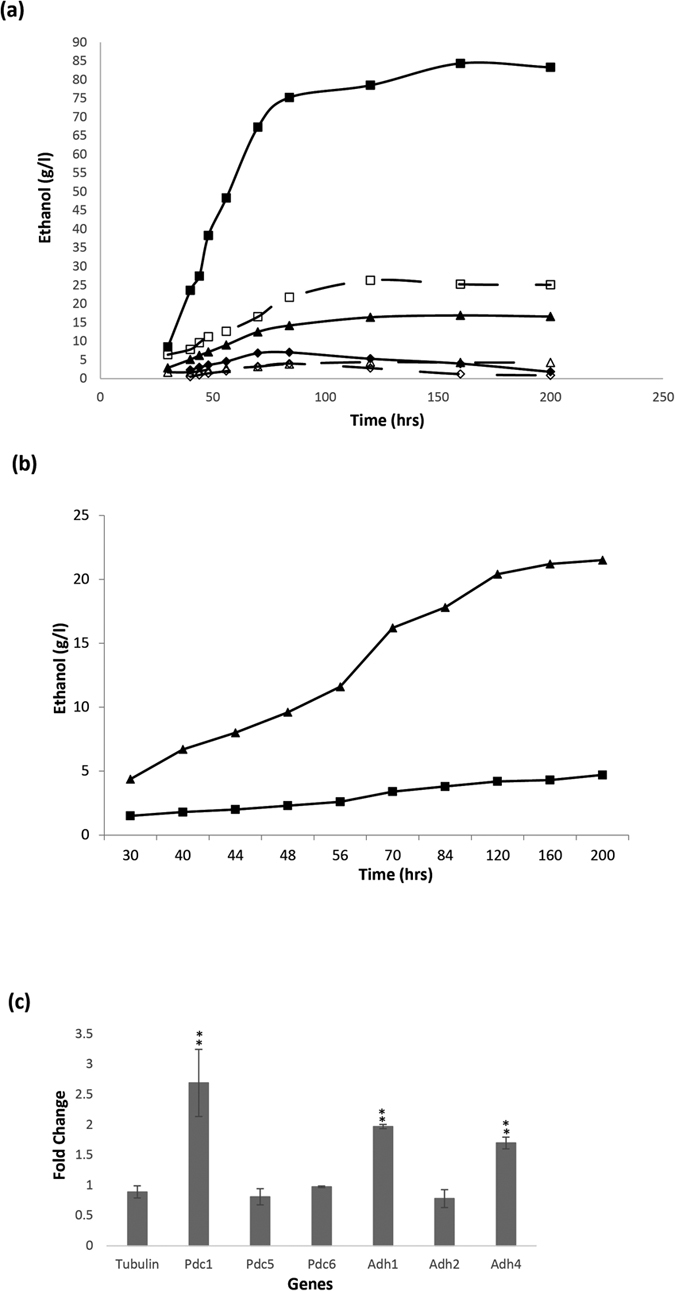
Expression of *AtMed15* in yeast increases ethanol production. (**a**) Ethanol concentration was measured at different time point in the media of yeast cells grown at 30 °C in different amount of glucose added to SD^−^trp. Ethanol concentration in AH109:vector yeast (–) is shown by ◊ (5% glucose), ∆ (10% glucose) and □ (20% glucose). Ethanol concentration in AH109:*AtMed15* Yeast (–) is shown by ♦ (5% glucose), ▲ (10% glucose) and ■ (20% glucose). (**b**) Ethanol concentration in the culture media of AH109:vector (■) or AH109:*AtMed15* (▲) yeast cells grown at 30 °C in the medium containing 8% of malt. (**c)** Real-Time PCR analysis of transcript level of ethanol pathway genes in AH109:vector and AH109:*AtMed15* cells. Expression of *Tub* was used for comparative analysis. Fold change represents expression level in AH109:*AtMed15* as compared to AH109:vector. For, statistical analysis, student’s t-test was performed to determine the significance of difference. **indicate significance difference at p < 0.01.

*Saccharomyces cerevisiae* and its related yeast strains are used in the production of alcoholic beverages. Nowadays, these yeast cells are also used for the production of bio-ethanol^47^. In order to know the effect of AtMed15-induced flocculation on the fermentation efficiency of yeast cells, ethanol concentration in the broth medium were measured after regular interval of time during growth period of yeast culture. Surprisingly, in both small scale flask culture and large scale batch culture in a fermentor, concentration of ethanol was found to be significantly more in the medium of *AtMed15* containing yeast cells in comparison to that of vector control during logarithmic phase of growth (Fig. S7). Next, we grew yeast cells in high percentage of sugar (5%, 10% and 20% glucose) and found almost four times more ethanol production by AtMed15 expressing yeast cells in comparison to the cells harboring just the vector (Fig. 6a). Generally malted grain or malt extract is used in the breweries. So we tested fermentation efficiency of AtMed15 containing yeast cells in 8% malt extract and again found four times more ethanol production in comparison to the vector control (Fig. 6b). Expression of *AtMed15* in yeast cells resulted in increased transcription of *Pdc1, Adh1* and *Adh4* (Fig. 6c). Thus, AtMed15-induced flocculation did not compromise the ethanol production but actually increased it by four times, and so can be proposed to be applied in manufacturing of alcoholic beverages and production of bio-ethanol.

## Discussion

Mediator complex is an important component of eukaryotic transcriptional machinery. A number of *in silico* analyses have shown presence of all the Med subunits in almost all type of eukaryotes across different kingdoms. One subunit, Med15, which is present in the tail module of Mediator complex has been found to be very important for transcriptional regulation of many processes in yeast and metazoans. In *Arabidopsis*, AtMed15 was first detected and identified in the biochemically purified Mediator complex from the suspension culture of *Arabidopsis*^28^. We did not find much of the primary sequence similarity in AtMed15 and other characterized Med15 proteins, but observed little homology in the amino terminal KIX domain (Fig. S1). Thus, not surprisingly, the *AtMed15* cDNA could not complement the functions of *Gal11* (yeast *Med15*) in galactose metabolism and multidrug resistance (Fig. 1). However, expression of *AtMed15* in yeast enhanced the adhesive nature of cells and induced flocculation in the culture (Fig. 2). Among the five yeast strains used in this study, the effect was more pronounced in Mat a strains in comparison to that in Mat α strains (Figs 2 and S3). Flocculation and adhesion in yeast cells are caused by flocculin/adhesin proteins including Flo1, Flo5, Flo9, Flo10, Flo11, Aga1, and Fig. 2. In *Saccharomyces cerevisiae* and *Schizosaccharomyces pombe*, mutation or deletion of genes coding for subunits of Cdk8 kinase module of Mediator complex, caused flocculation by increasing the transcript level of some flocculin/adhesin genes like Figs 1 and 2 and *Aga2*^48^,^49^,^50^. It is evident from gene expression analysis and phenotypic analysis of mutants that the subunits of kinase module function antagonistically to the subunits of tail module especially Med15^49^,^50^. In accordance to this we found that deletion of *Gal11*/*Med15* decreased transcript level of some of the flocculin genes like Figs 1 and 2 and *Aga2* (Fig. S4). In addition, deletion of *Gal11* also affected expression of *Aga1*. Thus, in yeast, Gal11 is required for transcriptional regulation of selected flocculin/adhesion genes. Based on the known mechanisms of gene regulation by Gal11, it is plausible to think that transcription regulators that regulate flocculation, might be targeting Gal11 for their transcriptional activity. It will be interesting to see how these regulators of flocculation interact with Gal11. Expression of AtMed15 in yeast caused enhanced stickiness of yeast colonies on agar surface, increase in cell-cell adhesion resulting into clump formation and flocculation of yeast cells in liquid culture by increasing the transcript levels of few *Flo* genes like *Flo1, Aga1* and *Flo11/Muc1*, suggesting that AtMed15 could partially complement function of Gal11 in regulation of flocculin/adhesin genes in yeast.

Flocculation can be affected by the genetic background of the strain in regards to flo genes i.e. Flo1 or Newflo type as Flo1 type strains are constitutively flocculant and Newflo type strains flocculate only in stationary phase^51^,^52^. Physiological factors also affect the flocculation, and the efficiency of physical factors affecting the flocculation again depends on the strain involved^37^. Generally flocculation is maximal at pH range between 2.5–5 though it varies with the type of Flo protein involved in flocculation. For instance Flo1-mediated flocculation is stable from pH 1.5 to 10^52^. AtMed15-induced flocculation was stable in pH range 1–8 (Fig. 3c). Flo1-mediated flocculation is known to be stable at a wide range of temperature^52^. Expression of AtMed15 in yeast induces expression of *Flo1* and so flocculation caused by it is very robust and unaffected by wide fluctuation of pH (1 to 8) and temperature (10–50 °C) (Fig. 3d). Expression of *Aga1* and *Flo11/Muc1* might also contribute to the robustness of AtMed15 conferred flocculation in yeast culture. The Lectin theory of flocculation explains that in the presence of calcium, cell wall bound lectins/flocculins bind to mannose moieties of cell wall of neighbouring cell to form flocs^35^,^36^,^38^. The AtMed15-induced flocculation showed maximum sensitivity to the mannose as compared to glucose, sucrose, maltose and fructose (Fig. 3f). This is because of the competition between mannose in the media and the mannose present in the wall of the yeast cells. Though glucose is a structural analogue of the mannose, higher concentrations of glucose did not affect AtMed15-induced flocculation. This is similar to previous observation of glucose being ineffective in the Flo1-mediated flocculation^53^,^54^. Thus requirement of mannose but not glucose confirms that AtMed15-induced flocculation in yeast culture is primarily mediated by expression of *Flo1* gene.

Yeast cells, especially *Saccharomyces cerevisiae*, are used in the production of alocoholic beverages, production of bio-ethanol, and in the applications like waste treatment. In all these applications, after the yeast has fulfilled its respective function, cells are separated from the broth in the downstream processing. The flocculation phenomenon is exploited in such applications for an easy, convenient and economical way to separate the flocs or aggregated yeast cells. A lot of efforts have been made and are being made to improve the process of flocculation. Up to now, other than natural selection of more flocculent strains, the improvement strategies involved over-expression of individual yeast flocculin genes like *Flo1* or *Flo11*^37^,^55^,^56^. According to our knowledge, this is the first report where a cDNA from other kingdom, *AtMed15* from *Arabidopsis*, has been used to induce flocculation in yeast. Importantly, expression of *AtMed15* caused increase in transcript level of more than one *Flo* gene (*Flo1, Flo11* and *Aga1*) in yeast. The AtMed15-induced flocculation in yeast is constitutive and robust as flocculation was observed throughout the cell growth, not affected by wide fluctuation of pH and temperature, and is immune to presence of different carbon sources (Fig. 3). These characteristics can make AtMed15-driven flocculation suitable for exploitation in bioreactors as the cells are self-immobilized in flocs/pellets.

Interestingly, the AtMed15-induced flocculation in yeast does not compromise with ethanol production. In yeast, ethanol is produced mainly from pyruvate, an intermediate product of carbohydrate metabolism, by two step process involving pyruvate decarboxylase (Pdc) and alcohol dehydrogenase (Adh). In *S. cerevisiae*, there are three genes *Pdc1, Pdc5* and *Pdc6*, that code for pyruvate decarboxylases^57^. In yeast cells carrying AtMed15, expression of *Pdc1, Adh1 and Adh4* was significantly higher resulting into production of more ethanol (Fig. 6c). The laboratory yeast strains including AH109 used in this study are not good ethanol producer. So these strains as such cannot be very useful in the industries. In this study we have used the laboratory strains just to demonstrate a novel strategy and presented a ‘proof-of-concept’ of utilization of AtMed15-induced flocculation in biotech industries as AtMed15-induced flocculation does not have any adverse effect on growth and fermentation efficiency. In fact, it actually increased the ethanol production during the course of cell growth and fermentation (Fig. 6). We suggest that AtMed15-driven flocculation accompanied with increased ethanol production can be exploited for use in bio-ethanol production, and brewery and beverage industries.

## Material and Methods

### Strain, media and culture conditions

Seedlings of *Arabidopsis thaliana* ecotype Columbia (col 0) were grown in culture room conditions at 22 ± 2 °C under long day light photoperiod. The *Escherichia coli* strain DH5α was used as a host for all plasmid construction and maintenance. All yeast strains used are listed in Table S1. All untransformed strains were routinely cultured in YPD medium composed of 10 g/l yeast extract, 20 g/l each of Glucose and peptone, at 30 °C and 150 rpm. While the pGBKT7 transformed yeast strains were grown on SD^−^trp (drop out media) containing 2% glucose, 6.67 g/l of nitrogenous base and 1.92 g/l of amino acid residues lacking tryptophan, the pGHM transformed cells were grown in SD^−^leu (leucine drop out media) with similar glucose and other nitrogenous base composition. Equal OD of cells were inoculated for every study. To study growth curve samples were collected after every 6 h till 30 h.

### Construction of gene expression cassettes

All the PCR primers used in the study are listed in Table S2. *AtMed15* full length CDS was amplified using *AtMed15*-F and *AtMed15*-R primers. Generally, a PCR condition of an initial denaturation at 94 °C for 3 min, followed by 30 cycles at 94 °C for 20 sec, 57 °C for 45 sec, 68 °C for 4 min and a final extension at 68 °C for 10 min. was used with some optimization to amplify DNA fragments. pGHM vector was made from pGADT7 vector by replacing smaller *Hind* III fragment with newly designed MCS and keeping the ADH promoter intact. The newly designed MCS was CATATGGCCATGGAGGCCGAATTCCCGGGGATCCGTCGACCTGCAGCGGCCGCATAA which had *NdeI, NcoI, SfiI, EcoRI, SmaI, BamHI, SalI* and *PstI* restriction sites. The amplified *AtMed15* was digested with *BamHI* and *EcoRI* and then ligated to pGBKT7 or pGHM vector generating the recombinant plasmid pGBKT:*AtMed15* or pGHM:*AtMed15*. All the strains used in this study are listed in Table S1 and the plasmids are given in Table S3.

### Microscopic analysis

For SEM analysis cells grown for 24 h were pelleted down, washed with 1× PBS and placed on grid. To make the cell surface conductive it was coated with palladium, in vacuum, using sputter coater. Scans were taken at different magnifications and resolutions. For wall study yeast cells were stained with Calcofluor at final concentration of 0.1mg/ml for 10 min. before washing with 1× PBS three times and then observed under confocal microscope.

### Flocculation Assay

The flocculation of yeast cells was quantitatively evaluated in 25 ml culture tubes having 10 ml of SD^−^trp media, incubated at 30 °C and 150 rpm. The whole culture was vortexed for 1 min and 1 ml of it was sampled to measure OD_600_, which was designated as ‘F1’. The tube was rested for 5 min and again 1 ml of supernatant was sampled to measure OD_600_ which was designated as ‘F2’. Flocculation ability (F) was calculated using following equation.


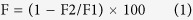


For adhesive growth assays, yeast strains were grown on YP agar medium (1% yeast extract, 2% bactopeptone) containing 2% of different carbon sources (glucose, sucrose, maltose and mannose) and incubated at 30 °C for 5 days. The plates were then rinsed with a thin layer of water. To study physiological parameters yeast cells grown at 30 °C, 150 rpm for 24 h were subjected to different treatments like for pH study, media was replaced with different pH media and for sugar sensitivity media was replaced with media having different sugars with different concentrations (0% to 10%) and grown for 8 h. To report temperature effect, sample was subjected to different temperatures ranging from 10 °C to 50 °C for 2 h.

### Gene expression analysis

Expression of flocculin/adhesion genes were studied by Microarray and Real-Time PCR. Total RNA was isolated from 5 ml of overnight grown yeast cultures, using RNeasy mini kit (Qiagen) as per instructions given in the manual. Quality of RNA was assessed on RNA Nanochip (Agilent) using 2100 Bioanalyzer (Agilent). High quality RNA was amplified and labeled with Cy3 using One Color Spike Mix (Agilent) and then used to hybridize G4813A Yeast Microarray slide (Agilent) as per manufacturer’s instruction. After washing, the arrays were scanned in Agilent Microarray Scanner G25665CA, and the strength of signals was analyzed by Feature Extraction Software (Agilent). After this the raw data were imported to GeneSpring GX 11.5.1 software (Agilent) for detailed analysis and heat map generation. For Real-Time PCR analysis, 5 μg of total RNA was used to prepare 33 μl of cDNA using oligo dT primers by first-strand cDNA synthesis kit (GE healthcare). The cDNA thus prepared was used as template for PCR analysis. *Actin* was used as internal control. We analysed transcript level of *Tubulin* as comparison control for the expression analysis of genes of interest. The reaction conditions were: (95 °C for 5 min, followed by 40 cycles at 95 °C for 30 min and 60 °C for 60 sec, and then a final extension of 72 °C for 5 min). Real-Time PCR was performed and analysed in 7900 HT Real-Time PCR (Applied Biosystem) detection system. At least three replicates were analyzed. The average of threshold cycle (Ct) value of each gene (in triplicate) was normalized to Ct value of *actin* (ΔCt = Ct target gene- Ct *actin*). This was further normalized (ΔΔCt) to reference sample (AH109) for calculating the fold change as 2^−∆∆Ct^). Statistically, the significant changes were analysed by student’s t-test at p value 0.01. The correlation between expression profile of the selected genes, measured by microarray and Real-Time PCR, was determined using Microsoft Excel tool.

The gene ontology annotations were performed on www.yeastgenome.org/cgi-bin/GO/goTermFinder.pI with default settings. For the experiments described here, we used only the “biological process” and “cell components” ontologies. Gene ontology (GO) classes with fewer than 50 or more than 250 genes represented in the data were not considered.

### Estimation of Ethanol

For the experiments in flask, yeast cells were grown in 250 ml culture media in 500 ml Erlenmeyer flask kept at 30 °C and shaking at 200 rpm. For growing cells in fermentor, medium was sterilized in fermentor (3L jacketed glass vessel) by autoclaving. The pH probe was calibrated before autoclaving the fermentor. To confirm that control and subject have same quantity of inoculum, yeast cells were deflocculated using 100 mM of EDTA and equal amount of cells were inoculated. For ethanol estimation, SD^−^trp media with 2%, 5%, 10% or 20% of glucose or 8% of malt was used as carbon source. The pH of medium was maintained at 6.5 ± 0.05 with automatic addition of 2N NaOH and 2N H3PO4. Sterilized Silicone antifoam was added (2–3 drops) to prevent excessive foaming. An Applikon AD101, a 3 L jacketed glass vessel with control unit was used for batch cultivation of cells for ethanol production. Aerobic condition was maintained by keeping dissolved oxygen (DO) level more than 20 percent and impeller speed of 800 rpm. DO probe was calibrated by one point calibration.

1 ml of ethyl acetate was added to the filtered sample, followed by 5 min of vortexing at maximum speed. Finally, the tubes were centrifuged to facilitate phase separation and the organic phase was subjected to gas chromatography. Ethanol estimation was done using gas chromatography. Rxi-1ms (RESTEK), non-polar, cross bond dimethyl polysiloxane capillary column was used. As direct injection of broth into such gas chromatography column is not recommended so for the safer use of column we performed organic extraction of ethanol using ethyl acetate^58^. Samples were collected at regular interval and filtered through 0.2 μm filter. In order to maintain the spirit of comparison, control samples and standards were subjected to exactly the same treatment. Conditions for ethanol estimation were standardized at constant pressure with a linear velocity of 33.6 ml/min having a split ratio of 1:50. A standard curve was generated, on the basis of peak area, using different concentrations (0.2%, 0.5%, 1%, 5%, 10% and 20%) of pure ethanol. Volume of ethanol in media was calculated from the standard curve using the straight line equation


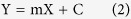


Mass of ethanol formed was calculated using the formula


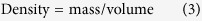


## Additional Information

**How to cite this article**: Dahiya, P. *et al*. Expression of AtMed15 of *Arabidopsis* in yeast causes flocculation and increases ethanol production in yeast culture. *Sci. Rep*. **6**, 27967; doi: 10.1038/srep27967 (2016).

## Supplementary Material

Supplementary Information
