# Supplementary material for: Expression of AtMed15 of Arabidopsis in yeast causes flocculation and increases ethanol production in yeast culture
Source: Sci Rep. 2016 Jun 16;6:27967. doi: 10.1038/srep27967 (PMC4910046; doi:10.1038/srep27967)
Supplement: Supplementary Information [file srep27967-s1.pdf]

Expression of AtMed15 of *Arabidopsis* in yeast causes flocculation and increases ethanol production in yeast culture

Pradeep Dahiya[1,2], Divya S. Bhat[1,2] and Jitendra K. Thakur[1#]
[1]National Institute of Plant Genome Research, Aruna Asaf Ali Marg, New Delhi 110067, India.

[2] These authors contributed equally

[#] Corresponding author
Email: jthakur@nipgr.ac.in
Phone: +91-11-26735221
Fax: +91-11-26741658

```
                                                     KIX-domain
HsMED15         ------------MDVSGQETDWRSTA---FRQKLVSQIEDAMRKAG--------VAHSKS
DmMED15         -----------------MTEDWQSQK---FRQNVISKIHDLLPPNA--------QDQTKN
AtMED15         MDNNNWRPSLPNGEPAMDTGDWRTQLPPDSRQKIVNKIMETLKKHLPFSGPEGINELRRI
CeMED15         ----------------MSEEDWPSPK---FREHVIQRL-EPELARNRQNAPN--LPVPGD
ScMED15         ----------MSAAPVQDKDTLSNAERAKNVNGLLQVLMDINTLNGGSSDT--ADKIRIH
                                    :: .       : ::. : :

                              KIX-domain
HsMED15         SKDMESHVFLKAKTRDEYLSLVA-RLIIHFRDIHNKKSQASVSDPMNALQSLTGGPAA--
DmMED15         AGVMENHIFRKSRTKDEYLGLVA-KLFMHYKDMS-RKSQQQQQQQQQQGGPPPNAEMG--
AtMED15         AARFEEKIFSGALNQTDYLRKISMKMLTMET--KSQNAAGSSAAIPAANNGTSIDSIPT-
CeMED15         ARQVEEYVFAKCMSKDEYMRTIA-KVINAINCN-SKSAAVPSVLQPSQFHSPP-------
ScMED15         AKNFEAALFAKSSSKKEYMDSMNEKVAVMRNTYNTRKNAVTAAAANNNIKPVEQHHINNL
                :  .*  :*  . .: :*:  :  ::.   .    :

HsMED15         GAAGIGMPPRGPGQSLGGMGSLGAMGQPMSLSG-QPPPGTSGMAPHSMAVVST-------
DmMED15         GGQNMMQDPLNALQNLASQGNRNPQMMPMGAGGGAPVPGGPGTASNLLQSLNQ-------
AtMED15         NQGQLLPGSLSTNQSQAPQPLLSQTMQNNTASGMTGSTALPSSMPPVSSITNNNTTSVVN
CeMED15         CTTAALLGNTPAGGTPGYRAPVPPDPQPTSAQARNPPVTVATTQASTTPSAPN-------
ScMED15         KNSGNSANNMNVNMNLNPQMFLNQQAQARQQVAQQLRNQQQQQQQQQQQQRRQLTPQQQQ
                              .                 .

HsMED15         --------------------------ATPQTQLQLQQVALQQQQQQQQFQQQQQAALQQQ
DmMED15         --------------------------QRPGQQQMQPMSNIRGQMPMG----AGGAGAQQM
AtMED15         QNANMQNVAGMLQDSSGQHGLSSNMFSGPQRQMLGRPHAMSSQQQQQPYLYQQQLQQQLL
CeMED15         ---------------------------PPGGLPAPSASATAAVAAAVASFPSPDTSIRPG
ScMED15         LVN------QMKVAPIPKQLLQRIPNIPPNINTWQQVTALAQQKLLTPQDMEAAKEVYKI
                                            *

HsMED15         QQ-----------------QQQQQQFQAQQSAMQQQFQAVV-------------------
DmMED15         MQ-----------------VQQMQQGGNAPGVMNVMGAGGG-------------------
AtMED15         KQNFQSGNVPNPNSLLPSHIQQQQQNVLQPNQLHSSQQPGVPTSATQPSTVNSAPLQGLH
CeMED15         GQ----------------ITPGSQAPGGGPTPAPNVPFPNG-------------------
ScMED15         HQQLLFKAR----------LQQQQAQAQAQANNNNNGLPQN------------------G
                 *                     *

HsMED15         --QQQQQLQQQQQQQQHLIKLHHQNQQQIQQQQQQLQRIAQLQLQQQQQQQQQQQQQQQQ
DmMED15         --QNQGQIVGNPGQQMGVGVGMPNQMVGPGPNSGPAVGGAGGPNAAPGAGGPGPNQMQGG
AtMED15         TNQQSSPQLSSQQTTQSMLRQHQSSMLRQHPQSQQASGIHQQQSSLPQQSISPLQQQPTQ
CeMED15         ----SSQMNGGPAMGQPPPQMGAPNMGGPPNGYGGYGMMNGPPGSGAPMGGNPYNQQIKK
ScMED15         NINNNINIPQQQQMQPPNSSANNNPLQQQSSQNTVPNVLNQINQIFSPEEQRSLLQEAIE
                    .                                                  *

HsMED15         ALQAQPPIQ-----------------------------------------QPPMQQPQPP
DmMED15         PMNVNAMQQ-----------------------------------------MPPMQQIQ--
AtMED15         LMRQQAANSSGIQQKQMMGQHVVGDMQQQHQQRLLNQQNNVMNIQQQQSQQQPLQQPQQQ
CeMED15         DMDQARPWD---------------------------------------------------
ScMED15         TCKNFEKTQLGSTMTEPVKQSFIRKYINQKALRKIQALRDVKNNNNANNNGSNLQRAQNV
                        .

HsMED15         PSQALPQQLQQMHHTQHHQPPPQPQQPPVAQNQPSQLPPQSQTQPLVS------------
DmMED15         -QNQLGMGMNPMMRMGQGNGMGGPQGMPGQGMQGMPQGPHNVVGGPAG------------
AtMED15         QKQQPPAQQQLMSQQNSLQATHQNPLGTQSNVAGLQQPQQQMLNSQVGNSSLQNNQHSVH
CeMED15         -PSAHMYQQQPQWGAMPPQQPHGYPGRPMNGQGATPTGPSSVLESLIN------------
ScMED15         PMNIIQQQQQQNTNNNDTIATSATPNAAAFSQQQNASSKLYQMQQQQQ----------AQ
                  .      :                 .

HsMED15         ---------QAQALPGQMLYTQPPLKFVRAP------------MVVQQPPVQPQVQQQQT
DmMED15         ---------QQQVG-GAGLPPNAVQQGGMNP------------MGGMGVNMPPNLQQK-P
AtMED15         MLSQPTVGLQRTHQAGHGLYSSQGQQSQNQPSQQQMMPQLQSHHQQLGLQQQPNLLQQDV
CeMED15         ---------QPQQYPGHHNQMGPPGD-----------------RNVAAQRAAAQQQQQQQ
ScMED15         AQAQAQAQAQAQAQAQAQAAQAAQAQAQAQAQAQAQAQAQAQAQAQAQAQAQAQAQAHAQ
                         *               .                          .:   :

HsMED15         AVQTAQAAQMVAPGVQMITEALAQGGMHIRARFPPTTAVSAIPSSSIPLGR---------
DmMED15         NMPMGQAGQMFPG---------NRGGVGVGGQQPGQPFMRSSPSPADAQQL---------
AtMED15         QQRLQASGQVTGSLLPPQNVVDQQRQLYQSQRTLPEMPSSSLDSTAQTESANGGDWQEEV
CeMED15         QQQQQQRPGMVPN----------QGMMSSEDQTVYSAKLRNMRGSCDSLRTR--------
ScMED15         HQPSQQPQQAQQQPNPLHGLTPTAKDVEVIKQLSLDASKTNLRLTDVTNSLS--------
                          :    :            .  .

HsMED15         --------------------QPMAQVSQSSLPMLS-------------------------
DmMED15         --------------------QQQAQLQQMQQ-----------------------------
AtMED15         YQKIKSMKETYLPDLNEIYQRVAAKLQQDSMPQQQRSDQLEKLRQFKTMLERMIQFLSVS
CeMED15         --------------------ARQCRHEGNHEAAHK-------------------------
ScMED15         ---------------NEEKEKIKMKLKQGQKLFVQVSN----------------------
                                        : .

HsMED15         -------------------------------------SPSPGQQVQTQPSMPPPPQPSPQ
DmMED15         ----------------------------------------QQQQLVVGNQTPTQQPPTPQ
AtMED15         KSNIMPALKDKVAYYEKQIIGFLNMHRPRKPVQQGQLPQSQMQPMQQPQSQTVQDQSHDN
CeMED15         ---------------------------------------LEVMLSVLEGKRVVSLEYLNH
ScMED15         --------------FAPQVYIITKNENFLKEVFQLRIFVKEILEKCAEGIFVVKLDTVDR
                                                                           .
```

```
HsMED15         PGQPSSQPNSNVSSGPAPSPSSFLPSPSPQPS-QSPVTARTPQNFSVPSPGP--------
DmMED15         MPTPNMIPSPALVPQSSPQMMQMQNSQRNIRQ-QSPSAS-----INTPGQVT--------
AtMED15         QTNPQMQSMSMQGAGPRAQQSSMTNMQSNVLS-SRPGVSAPQQNIPSSIPASSLESGQGN
CeMED15         LEMWIARKQDFLNIAPMSQNQNHMGMNDPMMN-GEHAMLGNGQVPNPYGGHP--------
ScMED15         LIIKYQKYWESMRIQILRRQAILRQQQQMANNNGNPGTTSTGNNNNIATQQN--------
                                               .

HsMED15         --------------------------------------LNTPVNPSSVMSPAGSSQAEEQ
DmMED15         --------------------------------------GNSPFNPQ-----------EEA
AtMED15         TLNNGQQVAMGSMQQNTSQLVNNSSASAQSGLSTLQSNVNQPQLSSSLLQHQHLKQQQDQ
CeMED15         ---------------------------------------GYGHQQYMGPPPPHMQMHQPP
ScMED15         --------------------------------------MQQSLQQMQHLQQLKMQQQQQQ
                                                                         :

HsMED15         QYLDKLKQLSKYIEPLRRMINKIDKNE-----DRKKDLSKMKSLLDILTDPSKRCP----
DmMED15         LYREKYKQLTKYIEPLKRMLAKISNDG-----TNVEKMTKMSKLLEILCNPTQRVP----
AtMED15         QMQLKQQFQQRQMQQQQLQARQQQQQQQLQARQQAAQLQQMNDMNDLTSRQGMNVSRGMF
CeMED15         MWHQQQHQQQQRMMPQDHMMMQGGGGPVHGMYRGDMGHDPMTSPVNNHRHAPYPNPAAMR
ScMED15         QQQQQQQQQQQQQQQQQHIYPSSTPGVANYSAMANAPGNNIPYMNHKNTSSMDFLNSMEN
                    : :   :          .                  :    .

HsMED15         ---------------------------------------------LKTLQKCEIALEKLK
DmMED15         ---------------------------------------------LETLLKCEKALEKMD
AtMED15         QQHSMQGQRANYPLQQLKPGAVSSPQLLQGASPQMSQHLSPQVDQKNTVNKMGTPLQPAN
CeMED15         N--------------------------------------------NMRMPNGPGPIGRDR
ScMED15         TPKVPVSAAATPSLNKTINGKVNGRTKSNTIPVTSIPSTNKKLSISNAASQQPTPRSASN
                                                                  :   .

HsMED15         NDMAVPTPPPPPVPPTKQQYLCQP------------------------------------
DmMED15         ---------LISYSGQQFGKSSNP------------------------------------
AtMED15         SPFVVPSPSSTPLAPSPMQVDSEKPGSSSLSMGNIARQQATGMQGVVQSLAIGTPGISAS
CeMED15         NSMSGSSMSGPSSGAPSMNPMGTP------------------------------------
ScMED15         TAKSTPNTNPSPLKTQTKNGTPNPNN----------------------------------
                           .

HsMED15         ------------LLDAVLANIRSPVFNHSLYRTFVPAMTAIHGPPITAPVVCT-------
DmMED15         ------------LLEVINTTLQSPVANHTLYRTFRPTLELLFGTDITAPVPAK-------
AtMED15         PLLQEFTSPDGNILNSSTITSGKPSATELPIERLIRAVKSISPQALSSAVSDIGSVVSMV
CeMED15         ----------NQKMGTPGSMGGMSGLDDLNYDDFLPNPTPIDALQPTLHVGQN-------
ScMED15         ------MKTVQSPMGAQPSYNSAIIENAFRKEELLLKDLEIRKLEISSRFKHRQEIFKDS
                             :                   :      :     :  .

HsMED15         ------------------------------------------------------------
DmMED15         ------------------------------------------------------------
AtMED15         DRIAGSAPGNGSRASVGEDLVAMTKCRLQARNFMTQEGMMATKKMKRHTTAMPLSVASLG
CeMED15         ------------------------------------------------------------
ScMED15         PMDLFMSTLG--------------------------------------------DCLGIK

HsMED15         ------------------RKRRLEDDE--RQSIPSVLQGEVARLDPKFLVNLDPSHCSNN
DmMED15         ------------------RPRVEEKSTSFEQEVPHVLQGEIARLDTKFKVKLDTTSQINN
AtMED15         GSVGDNYKQFAGSETSDLESTATSDGKKARTETEHALLEEIKEINQRLIDTVVEISDDED
CeMED15         --------------------SMNAGPPVQRSNLNETARKELQILDARFEIDPNHQRHDAN
ScMED15         DEEMLTSCTIPKAVVDHINGSGKRKPTKAAQRARDQDSIDISIKDNKLVMKSKFNKSNRS
                                                       ::   : ::           .

HsMED15         GTVH---------------------------LICKLDDKDLPSVPPLELSVPADYPAQSP
DmMED15         KAIR---------------------------LICCLDDKRLPSVPPVSVSVPEEYPWQAP
AtMED15         AADPSEVAISSIGCEGTTVRFSFIAVSLSPALKAHLSSTQMSPIQPLRLLVPCSYPNGSP
CeMED15         HIIV----------------------------VCKIRNQQFP---PLRLVVPTTYPAGNV
ScMED15         YSIALSN-------------------------VAAIFKGIGGNFKDLSTLVHSSSPSTSS
                                                 . : .        :   *    *

HsMED15         LWIDRQWQYDANPFLQSVHRCMTSR-------LLQLPDKHSVTALLNTWAQSVHQACLSA
DmMED15         DCSLAEQEYSATPFLQTVQQALIAR-------ISKLPKNYSLSHLLDTWEMAVRQACSPQ
AtMED15         SLLDKLPVETSKENEDLSSKAMARFNIL----LRSLSQPMSLKDIAKTWDACARAVICEY
CeMED15         TVDRAVIDLDAYLYDDLQNSVYERLS---------RPGLSSITDYLNAWEEQVNQYQNQT
ScMED15         NMDVGNPRKRKASVLEISPQDSIASVLSPDSNIMSDSKKIKVDSPDDPFMTKSGATTSEK
                               :                    .   .:    ..:

HsMED15         A------------------------------
DmMED15         SKPRAVCELSTLLGV----------------
AtMED15         AQQFGGGTFSSKYGTWEKYVAAS--------
CeMED15         SGGLDVAFNVGNDFFYDNLNL----------
ScMED15         QEVTNEAPFLTSGTSSEQFNVWDWNNWTSAT
```

Figure S1: Alignment of amino acid sequences of Med15 from different eukaryotes. Residues identical in all the proteins are highlighted with black colour and marked with '*', whereas similar residues are highlighted in grey colour and marked with ':'. KIX-domain is marked with a black line on the top of the sequences. Hs *Homo sapiens*, Dm *Drosophila melanogaster*, At *Arabidopsis thaliana*, Ce *Caenorhabditis elegans*, and Sc *Saccharomyces cerevisiae*.

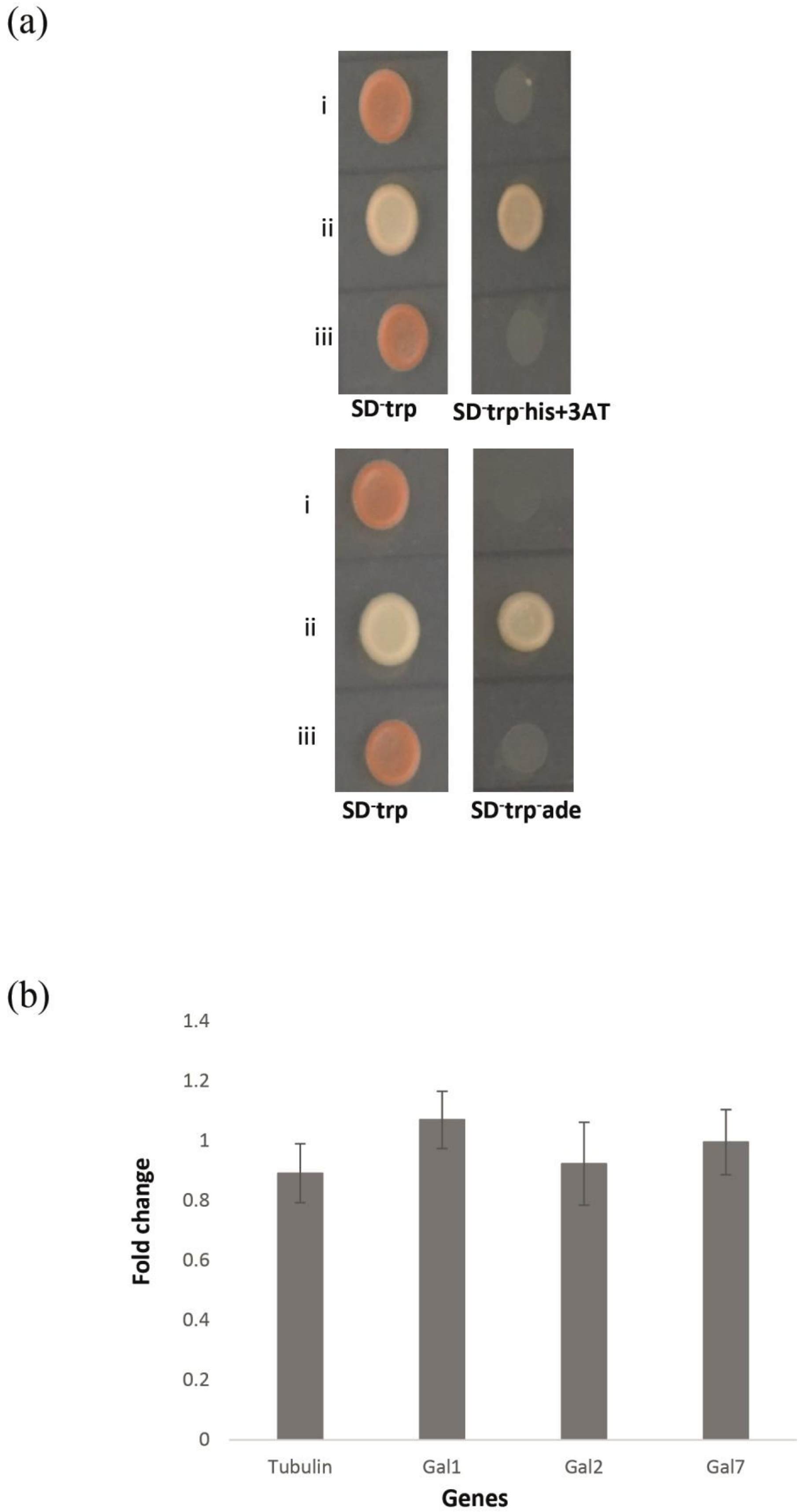

Figure S2: Activation of reporter genes by AtMed15. (**a**). Yeast colonies AH109:*pGBKT7* (i), AH109*:TFAD* (Transcription factor activation domain) (ii) and AH109:*AtMed15* (iii) were spotted on $SD^{-}trp$, $SD^{-}trp^{-}his+3AT$ and $SD^{-}trp^{-}ade$ to check activation of *His3* and *Ade2* . The plates were photographed after 72h. (**b).** Real-Time PCR analysis of transcript level of Gal4 target genes for AH109 yeast expressing AtMed15. *Actin* was used as internal quantitative and qualitative control. *Tubulin* was used as a comparison control for the expression analysis. Fold change represents expression level in AH109:*AtMed15* as compared to AH109:Vector.

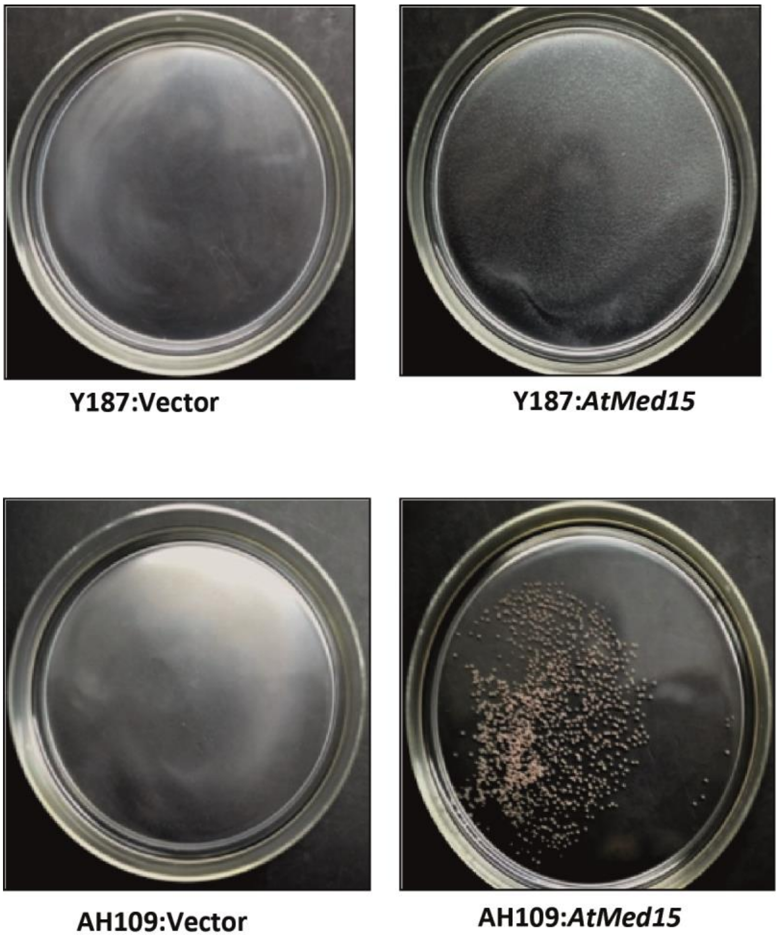

Figure S3: Cell-cell adhesion as visualized by clump formation in Mat a (AH109) and Mat α (Y187) strains. Overnight grown yeast cultures, Mat α (upper panel) and Mat a (lower panel) were transferred to petriplates, and rotated slowly for few hours to see cells clumps.

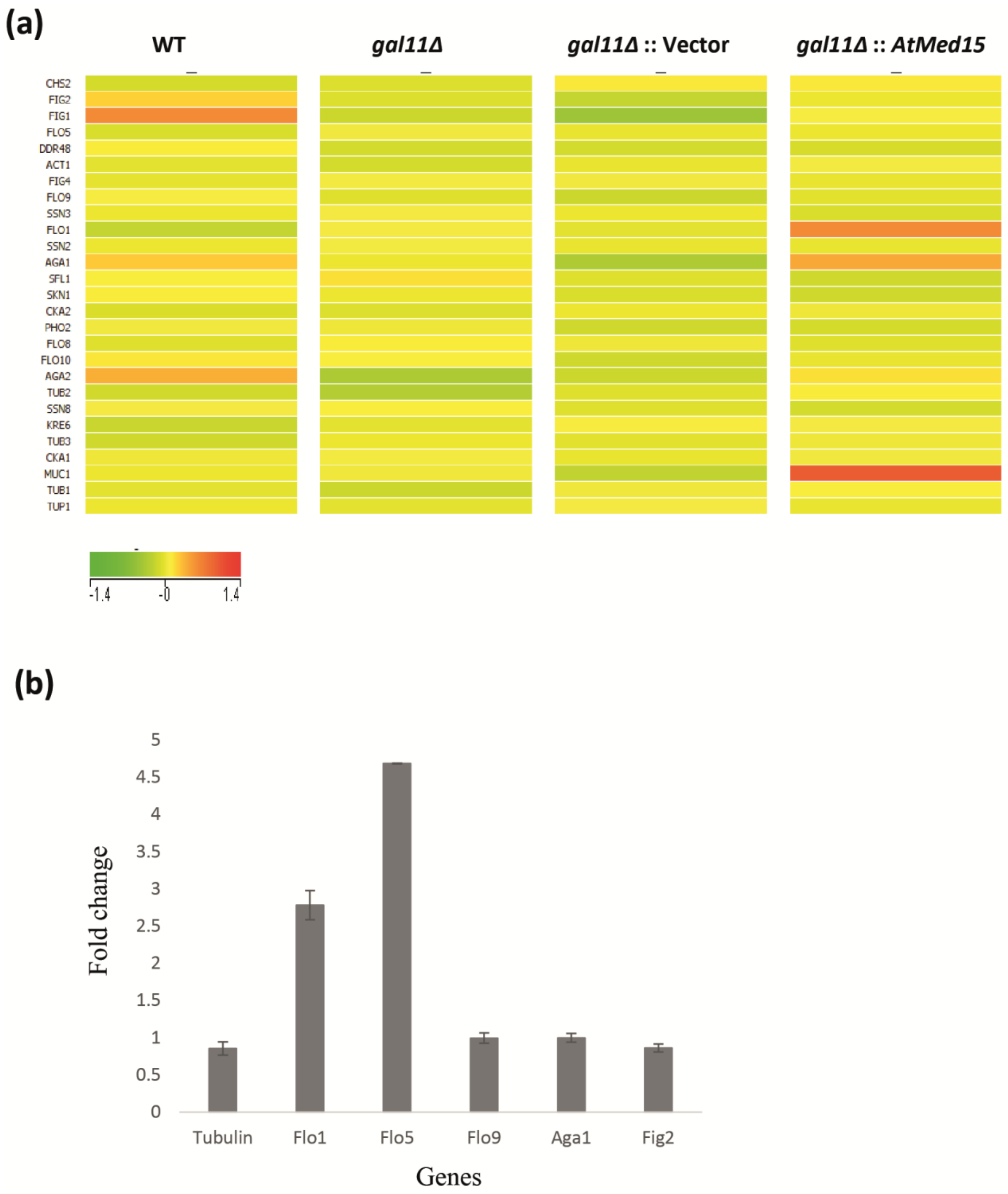

Figure S4: Expression of *AtMed15* increases transcript level of flocculin genes. (a). Microarray analysis to see the effect of AtMed15 on flocculation genes. Heat map of normalized and baseline transformed average $\log_2$ signal values of genes mentioned on the left, in the yeast strains wild type (WT), *gal11*Δ, *gal11*Δ:vector and *gal11*Δ:*AtMed15*. The colour legend is shown at the bottom. (**b).** Real-Time PCR analysis to study fold change in the transcript level of few selected flocculin/adhesin genes in the AH109:AtMed15 yeast cells as compared to AH109:Vector cells. *Actin* was used as internal quantitative and qualitative control. *Tubulin* was used as a comparison control for the expression analysis.

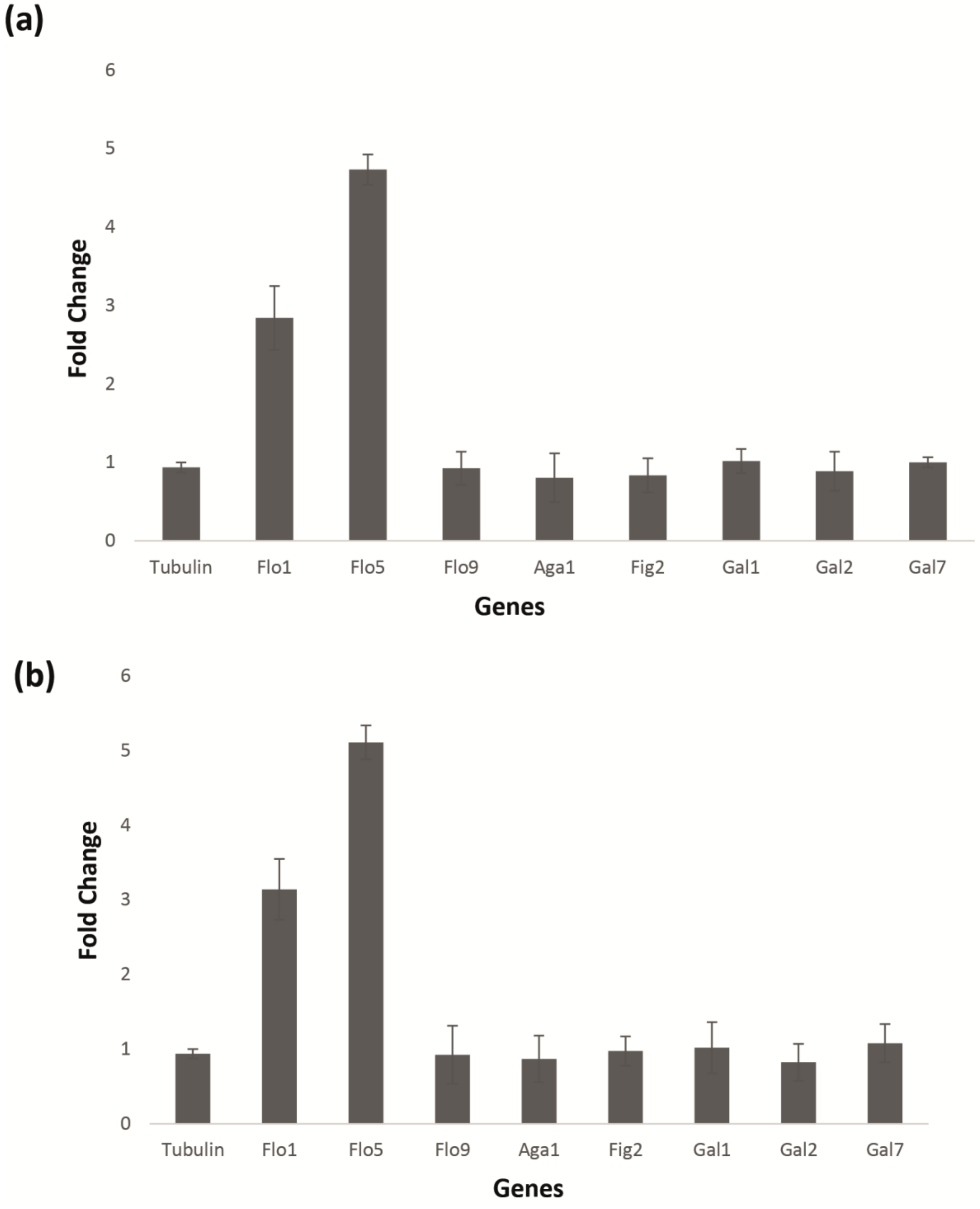

Figure S5: Effect of AtMed15 on the gene expression at different time points during the growth. (a) Real-Time PCR analysis to determine the fold change in the transcript level of few selected flocculin/adhesin and *Gal* genes in AH109:pGBKT7-*AtMed15* carrying yeast cells as compared to AH109:pGBKT7 cells after 12 h of growth. *Actin* was used as internal quantitative and qualitative control. *Tubulin* was used as a comparison control for the expression analysis. **(b).** Real-Time PCR analysis to see the fold change in the transcript level of few selected flocculin/adhesin and *Gal* genes in AH109:pGBKT7-*AtMed15* yeast cells as compared to AH109:pGBKT7 cell after 24 h of growth. *Actin* was used as internal quantitative and qualitative control. *Tubulin* was used as a comparison control for the expression analysis.

(a)

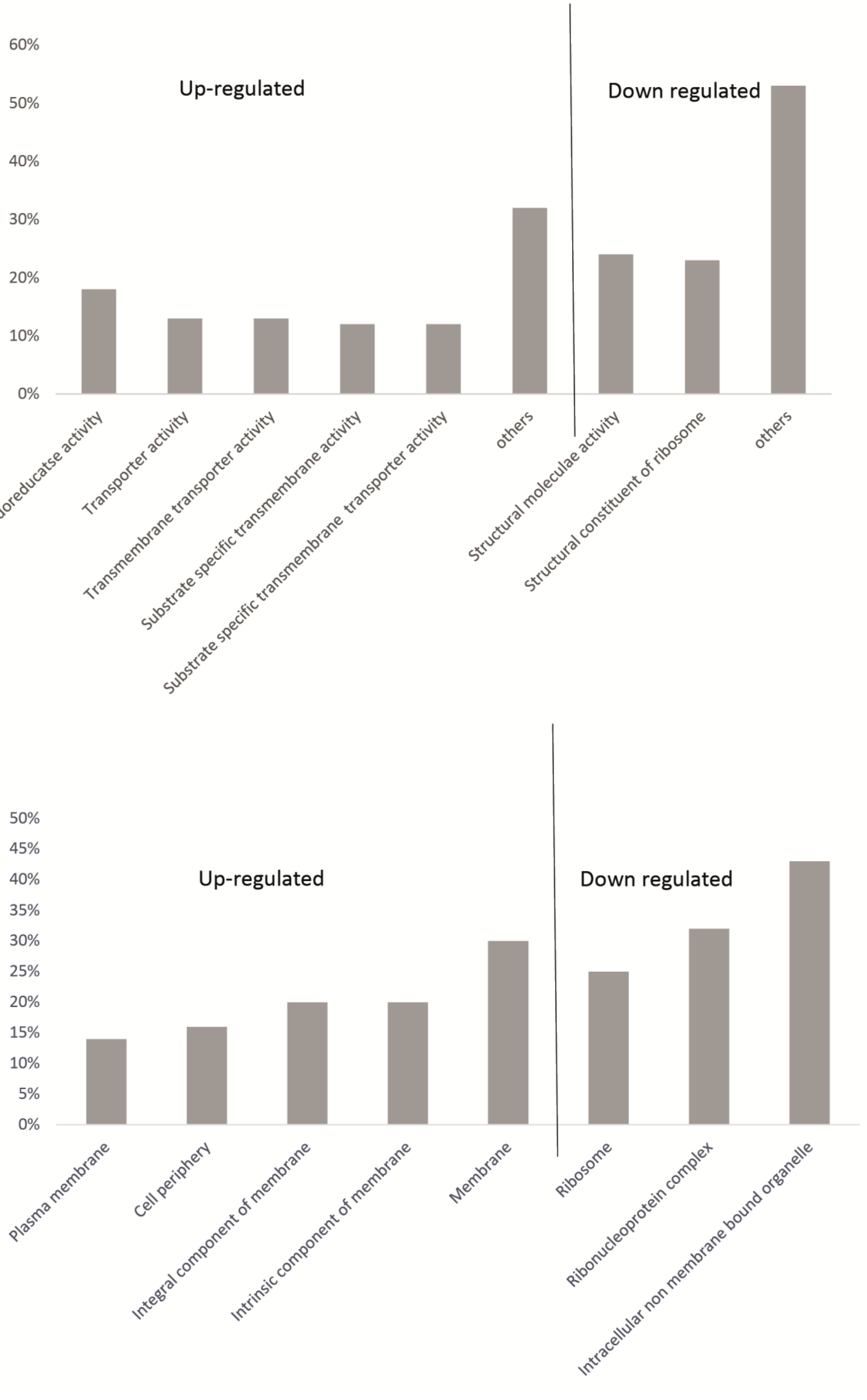

60%
50%
40%
30%
20%
10%
0%
Up-regulated
Down regulated
oxidoreducatse activity
Transporter activity
Transmembrane transporter activity
Substrate specific transmembrane activity
Substrate specific transmembrane transporter activity
others
Structural moleculae activity
Structural constituent of ribosome
others
50%
45%
40%
35%
30%
25%
20%
15%
10%
5%
0%
Up-regulated
Down regulated
Plasma membrane
Cell periphery
Integral component of membrane
Intrinsic component of membrane
Membrane
Ribosome
Ribonucleoprotein complex
Intracellular non membrane bound organelle

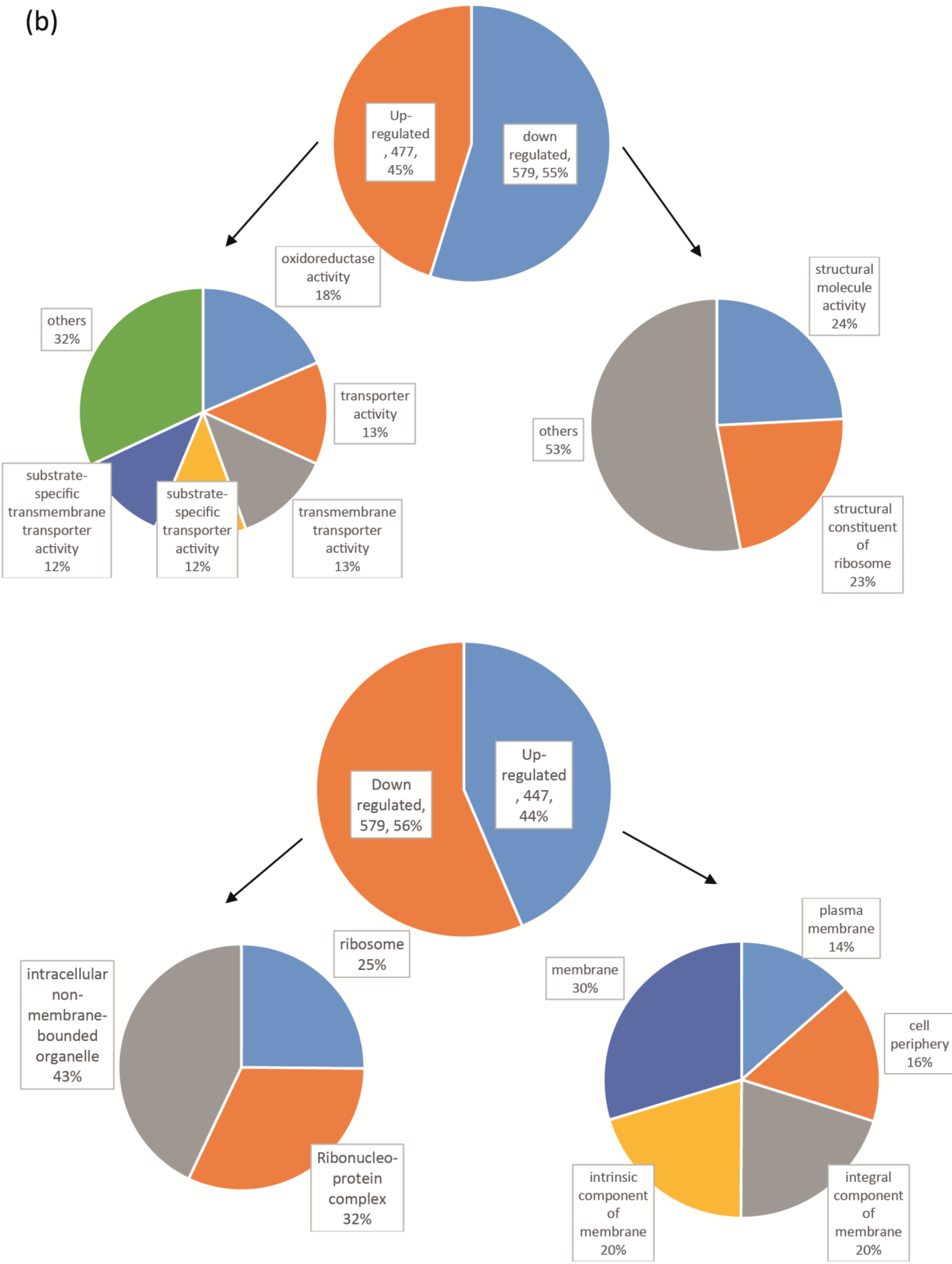

Figure S6: GO annotation of Microarray data of AtMed15 affected genes in AH109 yeast cells. (a) Bar chart representation of all the affected genes grouped on the basis of biological processes (upper panel) and on the basis of cellular components (lower panel). Maximally affected groups are shown for both up-regulated and down-regulated genes. **(b)**. Pie chart representation of all the affected genes arranged on the basis of biological processes (upper panel) and on the basis of cellular components (lower panel). Maximally affected classes are shown for both up-regulated and down regulated genes.

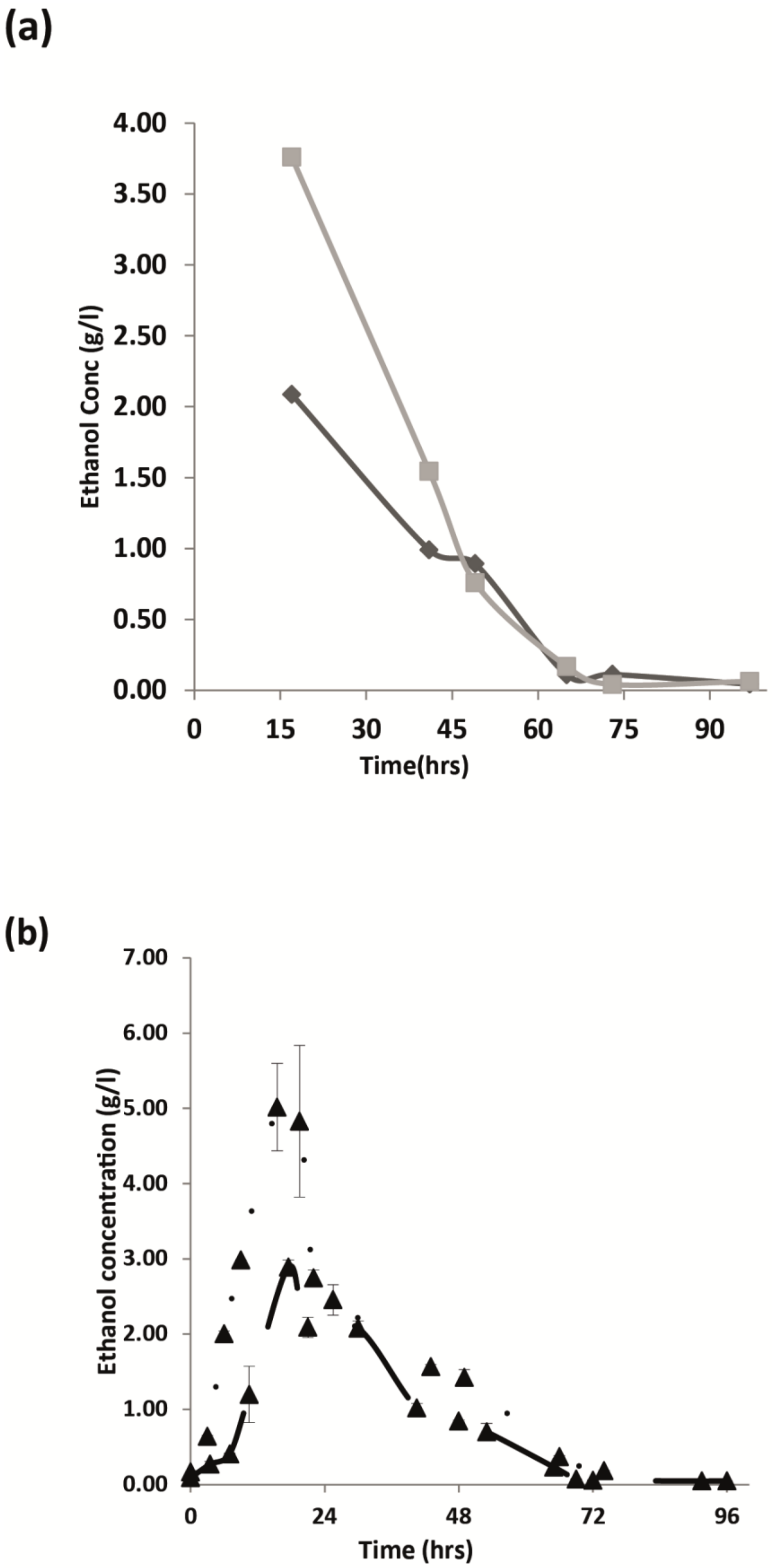

Figure S7: Ethanol production in small and large scale culture: (a). Equal amount of AH109 yeast cells carrying vector (■) or vector harboring *AtMed15* cDNA (■) were grown in 500ml of YPD broth in conical flask and then ethanol concentration was determined at different interval of time. **(b)**. Ethanol concentration was determined in culture of AH109:vector (**--▲--**) yeast cells and AH109 cells having AtMed15 (¨▲¨) in a batch fermentor (3 litres) at regular interval of time.

Table S1: Strains used in this study

Strain	Genotype	Source
BY4741	MATa *his*3Δ1 *leu*2Δ0 *met*15Δ0 *ura*3Δ0	(54)
BY4741 *gal11Δ*	BY4741 *gal11Δ*	(20)
AH109	MATa *trp*1-901*leu*2-3 112 *ura*3-52 *his*3-200 *gal*4Δ *gal*80Δ *LYS*2:: *GAL1*$_{UAS}$-*GAL*1$_{TATA}$-*HIS*3 *MEL*1*GAL*2$_{UAS}$-*GAL*2$_{TATA}$-*ADE*2 *URA*3:: *MEL*1$_{UAS}$-*MEL*1$_{TATA}$-*lac*Z	Clontech
JRY2334	MATa *ade*2-1 *his*3-11 15 *leu*2-3 112 *trp*1-1 *ura*3-1 *can*1-100	(55)
Y2HGOLD	MAT*a trp*1-901 *leu2*-3 112 *ura*3-52 *his*3-200 *gal*4 *Δ gal*80*Δ LYS2: : GAL*1$_{UAS}$–*Gal*1$_{TATA}$–*His*3, *GAL*2$_{UAS}$–*Gal*2$_{TATA}$–*Ade2 URA*3*:: MEL*1$_{UAS}$–*Mel*1$_{TATA}$ *AUR*1-*C MEL*1	Clontech
YI87	MATα ura3-52 *his*3-200 *ade* 2-101 *trp* 1-901 *leu2-3* 112 *gal*4Δ met$^-$*gal*80Δ *URA*3:: *GAL1*$_{UAS}$-*GAL*1$_{TATA}$-*lac*Z *MEL*1	Clontech
W303	MATα *ade*2-1 *ura*3-1 *his*3-11 15 *trp*1-1 *leu*2-3 112 *can*1-100	(54)
SEY6210	MATα *leu2-3, 112 ura3-52 his3-200 trp1-901 Ade2-101 suc2-9; GAL*	*(56)*

Table S2: List of primers used in this study

**Cloning (5'-3')**	
***AtMed15*F**	GCGAATTCATGGATAATAACAATTGGAGG
***AtMed15*R**	GCGGATCCATTCAGGAAGCTGCTACATC
**Deletion Analysis primers (5'-3')**	
***AtMed15*_1_1080BamHI_R**	ATGGATCCGCATTAATTGTGTAGGC
***AtMed15*_1_2075BamHI_R**	ATGGATCCTTGCTGTACTGGCTTCC
***AtMed15*_1_3160BamHI_R**	TAGGATCCGGCTCTAATAAGGCGTTC
***AtMed15*_1_1060EcoRI_F**	TAGAATTCCAGCCTACACAATTAATGCG
***AtMed15*_1_2074EcoRI_F**	TAGAATTCGGGCAGCTTCCGCAATC
***AtMed15*_1_3142EcoRI_F**	TAGAATTCGAACGCCTTATTAGAGCCG
**RT-PCR (5'-3')**	
Flo1*-RTF**<br>Flo1*-RTR**	CGAAACTGGCAACACCAAGA<br>ACGAGGCTGTTGCGACATAGT
Flo9*-RTF**<br>Flo9*-RTR**	GGGTTCTTACACATTCAAGTTTGCT<br>GCAATGCTACCACCGACTGA
Flo5*-RTF**<br>Flo5*-RTR**	GGCAGTGCCAACAGCTTACTG<br>AGCAATAAGGACGCAATGAAGAC
Flo8*-RTF**<br>Flo8*-RTR**	TTCAGCCCTCATCCAATGTGGGTA<br>ATCCGGTCCTTGGTCTTCAACCAT
Fig2*-RTF**<br>Fig2*-RTR**	ACGGAGGCAGTAGAGGTGACA<br>ATAACAGTCGGTGTGCAAATCG
Act1*-RTF**<br>Act1*-RTR**	TCGTTCCAATTTACGCTGGTT<br>CGGCCAAATCGATTCTCAA
Tub1*-RTF**<br>Tub1*-RTR**	AGGAGGACGCGGCTAATAATTA<br>TCGCCCAAAATTTCTCTACCA
Aga1*-RTF**<br>Aga1*-RTF**	TGCCTTGGCATCTGATCCA<br>CCCATTTGCATCGTTTGTCTT
Adh1*-RTF**<br>Adh1*-RTR**	GAAGGTGCCGGTGTCGTT<br>ACCGATCTTCCAGCCCTTAAC
Adh2*-RTF**<br>Adh2*-RTR**	CGCTCGGTGGTGAAGTATTCA<br>TTAACGACTGCGCTAACAATGTC
Adh4*-RTF**<br>Adh4*-RTR**<br>***Pdc1*-RTF**<br>***Pdc1*-RTR**	GGTGTTTGTAACGCTGTCTTGTTG<br>GGCCTTTGGACATTGCATGT<br>CACCCAAGATACGGTGGTGTT<br>GCAGATTCAACGGCTTCCTT
Pdc5*-RTF**<br>Pdc5*-RTR**<br><br>***Pdc6*-RTF**<br>***Pdc6*-RTR**	TTGGGACCACTTGGCCTTATT<br>GGTAGCAACTCTGTGGGTTTCG<br><br>TGACGCAATTCCCAGCTTTT<br>CTGGGATGCTGTTCATCTATTGAC

Table S3: Plasmids used in this study

Plasmid	Description	Reference
pGBKT7	*GAL*4 $_{(1-147)}$ DNA-BD, *TRP1*, kan^r, c-Myc epitope tag	Clontech
pGADT7	*GAL4* $_{(768–881)}$ AD, *LEU2*, amp^r, HA epitope tag	Clontech
pGBKT7-*AtMed15*	*AtMed15* $_{(1-1335)}$ (full-length CDS) in pGBKT7	This study
pGHM	*LEU*2, amp^r, ADH1 promoter	This study
pGHM -*AtMed15*	*AtMed15* in pGHM	This study
pGHM -*eGfp*	*eGfp* in pGHM	This study
